# The world’s earliest Aral-Sea type disaster: the decline of the Loulan Kingdom in the Tarim Basin

**DOI:** 10.1038/srep43102

**Published:** 2017-02-27

**Authors:** Steffen Mischke, Chenglin Liu, Jiafu Zhang, Chengjun Zhang, Hua Zhang, Pengcheng Jiao, Birgit Plessen

**Affiliations:** 1 Faculty of Earth Sciences, University of Iceland, 101 Reykjavík, Iceland; 2 Institute of Mineral Resources, Chinese Academy of Geological Sciences, Beijing 100037, China; 3 MOE Laboratory for Earth Surface Processes, Department of Geography, College of Urban and Environmental Sciences, Peking University, Beijing 100871, China; 4 School of Earth Sciences and Key Laboratory of Mineral Resources in Western China, Lanzhou University, Lanzhou 730000, China; 5 Helmholtz Centre Potsdam, German Research Centre for Geosciences, Potsdam 14473, Germany

## Abstract

Remnants of cities and farmlands in China’s hyperarid Tarim Basin indicate that environmental conditions were significantly wetter two millennia ago in a region which is barren desert today. Historical documents and age data of organic remains show that the Loulan Kingdom flourished during the Han Dynasty (206 BCE–220 CE) but was abandoned between its end and 645 CE. Previous archaeological, geomorphological and geological studies suggest that deteriorating climate conditions led to the abandonment of the ancient desert cities. Based on analyses of lake sediments from Lop Nur in the eastern Tarim Basin and a review of published records, we show that the Loulan Kingdom decline resulted from a man-made environmental disaster comparable to the recent Aral Sea crisis rather than from changing climate. Lop Nur and other lakes within the Han Dynasty realm experienced rapidly declining water levels or even desiccation whilst lakes in adjacent regions recorded rising levels and relatively wet conditions during the time of the Loulan Kingdom decline. Water withdrawal for irrigation farming in the middle reaches of rivers likely caused water shortage downstream and eventually the widespread deterioration of desert oases a long time before man initiated the Aral Sea disaster in the 1960s.

The Tarim Basin (Xinjiang Province) in northwestern China hosts the Taklamakan Sand Sea, the second largest sand desert of the world. Precipitation in the basin is lower than 20 mm a^−1^ but streams from towering mountain ranges in the north, west and south flow along its margins and charged the closed-basin lake Lop Nur until the 1970s when the lake eventually desiccated^1^,^2^,^3^,^4^. According to historical documents of the Han Dynasty, the lake was once called “Puchang Sea” and estimated to have covered 17,000 to 50,000 km^2^ which places the Han-period lake in the range between today’s Lake Michigan (the 5^th^ largest lake on Earth) and Lake Balkhash (the 15^th^ largest lake;^5^). Ruins of ancient cities, remnants of fields, irrigation channels and roads have been found in the desert region^1^,^6^,^7^. Most of these cities existed in the northwest and west of the lake where the Tarim and Konqi Rivers flowed towards the ancient Lop Nur (Fig. 1). Loulan at the ancient Silk Road is among the most famous ruined cities, reported in historical documents almost 2000 years ago^8^ and re-discovered by the Swedish researcher and traveller Sven Hedin in 1899^1^. The flourishing Loulan Civilization was an important trade centre at the ancient Silk Road established at the beginning of the Han Dynasty^9^. Some of the well preserved mummies from the Tarim Basin are significantly older with ages up to 3800 years^10^, but most of the cities and farmlands were established during the first expansion of China’s mainland towards the west during the Han Dynasty^1^,^6^,^7^. This westward expansion of the Chinese Empire was accompanied by a large population migration and intensification of irrigation farming along the rivers in mountain forelands in the Tarim Basin and the Hexi Corridor (Gansu Province) farther to the east^1^,^5^,^11^. The expansion towards the west was enabled by stationed troops which not only secured the occupation but were used for the establishment of fortified cities and trade posts^12^,^13^. Even more important, the soldiers were the ones responsible for the large-scale reclamation of arable land and the establishment of irrigation farming in many regions which had not been intensively cultivated before^14^,^15^,^16^. Large-scale irrigation farming started in the Tarim Basin in the middle of the first century BCE^8^. According to the historical book by Ban Gu^8^, a total of 300,830 people lived in more than 20 kingdoms upstream of Lop Nur in the Tarim Basin including Loulan sometime during the Han Dynasty. Among them, the Loulan Kingdom included 14,100 inhabitants^8^. Agriculture was significantly intensified even in remote desert oases such as the lower Keriya and Niya River valleys where land use was restricted to the use of natural oases before^5^,^17^. Cultivated areas were significantly larger in the Tarim Basin during the Han Dynasty than during the Qing Dynasty (1644–1911) or even around 1950^5^. The extensive transformation of alluvial fans and plains to irrigated fields in the Tarim Basin and Hexi Corridor not only supported the migration of people from the previous mainland of the Chinese Empire during the Han Dynasty but led also to intensive trade along the Silk Road two millennia ago^1^,^2^,^5^,^6^,^7^,^12^. Historical documents describe diminishing river discharge and subsequent reductions in soldier’s grain rations after ca. 270 CE^1^,^18^. The cities had been abandoned when the monk Xuanzang travelled in the region in 645 CE^1^,^18^. Correspondingly, radiocarbon dating results for organic remains suggest that the abandonment of the cities started not earlier than 230 ± 66 CE^6^, leaving a period from ca. 200 to 645 CE for the decline of the Loulan Civilization. We investigated the sediments of Lop Nur and compared the results with palaeoclimate records from other lakes in the region to examine whether climate change triggered the deterioration of the flourishing desert oases suggested as result of previous studies^1^,^2^,^5^,^6^,^7^,^9^.

## Results and Discussion

A 5.7-m deep pit section in the centre of the Lop Nur Basin provides grain-size and stable isotope records for the past ca. 9000 years^19^,^20^. Poor to very poorly sorted sediments with a mean grain size in the silt fraction prevail apart from two layers of coarser sediments centered at 0.70 and 0.50 m depth (Fig. 2). The sand layers with grain size and shape similar to aeolian sands in the region were formed ca. 1800 and 1000 years ago (Fig. S1). The two layers of coarser sediments show partly better sorting than sediments beneath or above (Fig. 2) although both include a significant fraction of silt-sized material (39% for the lower and 14% for the upper layer)^20^. The lower layer of coarser sediments is underlain by a 0.20 m thick sequence of sediments with increasing mean grain sizes. The gradual increase from a mean grain size of fine silt at 0.90 m to fine sand at 0.70 m probably indicates a continuously increasing proportion of aeolian sediment accumulation and steady lake level decrease. In contrast, the upper layer of coarser sediments with a mean grain size of medium sand covers fine-grained sediments with a mean grain size of fine silt without transitional grain size change (Fig. 2). The observed abrupt grain size increase implies that the depositional setting either changed abruptly, or that transitional sediments with inverse grading were possibly accumulated at first during a gradual lake-level decline but were subsequently removed by wave-driven currents before the accumulation of coarser sediments. Soluble salt concentrations in the sediments beneath and in the two coarser layers are increasing and relatively high, respectively, and provide supporting evidence that the coarser sediment layers were accumulated during periods of low lake levels and more saline conditions in Lop Nur^20^.

The stable oxygen isotope values for ostracod shells are in the same range as the δ^18^O values for bulk carbonate from the same stratigraphic levels in the section (Fig. 2). Thus, the δ^18^O record for bulk carbonate represents a reliable record of the relative δ^18^O changes of past lake waters. The increasing δ^18^O values for bulk carbonate at 0.90 m depth indicate a rapid reduction of inflows to the lake and a salinity rise ca. 2300 years ago further amplified ca. 1000 years ago (Fig. 2). The drop of the δ^18^O values at 0.46 m probably reflects a higher discharge which entered Lop Nur again, and a recovery of the lake system for some time before the final desiccation in the last century. Earlier in the stable isotope record, the lowest δ^18^O values 8500 years ago indicate the wettest conditions in the Lop Nur region. The large increase at 2.00 m depth suggests a significant decrease in inflows and moisture availability ca. 7000 years ago. However, the stable isotope data from Lop Nur suggest that the lake level started to decline as early as 2300 years ago whilst the grain-size data indicate very low lake levels and near-desiccation periods starting ca. 1800 years ago. Sand-dominated sediments were not accumulated during the entire preceding period between 9000 and 1800 years which suggests that a fundamental change in the depositional environment occurred ca. 1800 years ago.

Accumulation of aeolian sand in the basin centre almost two millennia ago roughly corresponds to the time of reported grain-ration reductions in the Loulan Kingdom^1^ and the first abandonment of Tarim Basin cities inferred from radiocarbon dating^6^. Thus, Lop Nur apparently experienced lowest lake levels or possibly first periods of desiccation more or less during the time of the Loulan Civilization decline at the end of the Han Dynasty (220 CE) and afterwards.

**Figure 1:**
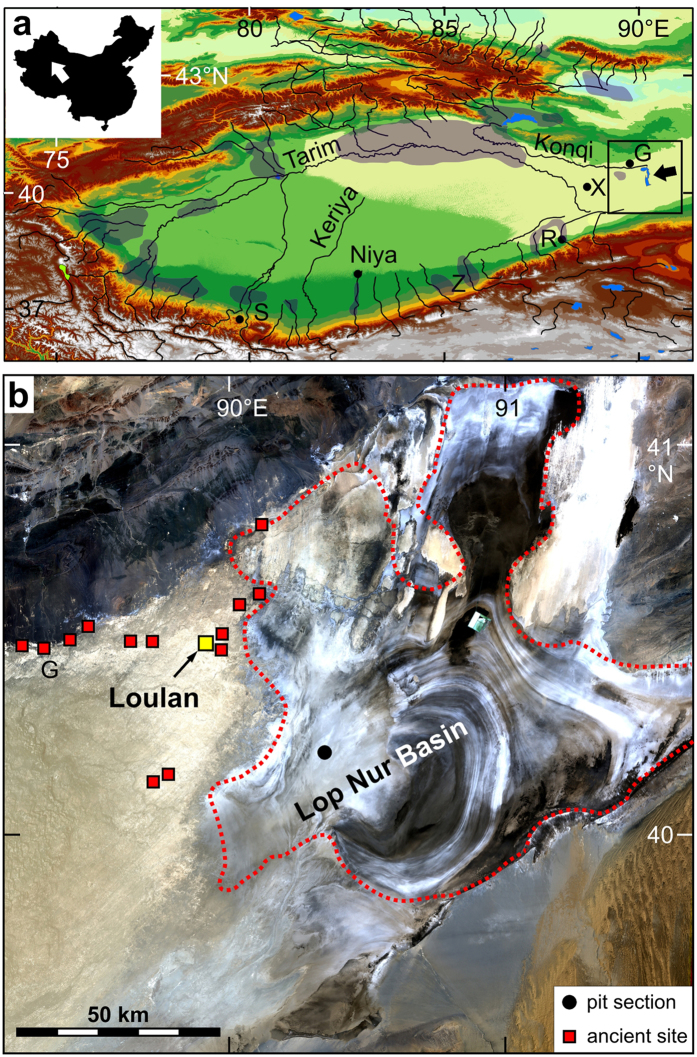
The ancient Lop Nur Lake in the Tarim Basin. (**a**) Tarim Basin with rivers, Lop Nur (black arrow) and ancient sites (S - Sampula, Z - Zaghunluq, R – Ruoqiang, X – Xiaohe, G – Gumugou), area of irrigated farmland during the Han Dynasty^1^,^5^ indicated by grey shading; inset shows location in China. Frame in the right indicates area shown in Fig. 1b. Background map was generated with data available from the Chinese Geospatial Data Cloud using ESRI’s ArcGIS (version 10.2; http://www.esri.com/). (**b**) The dry Lop Nur Basin with position of pit section (black dot) and Loulan and other sites. Ancient lake extent^1^ indicated by red dotted line. Background map generated with ESRI’s ArcGIS (version 10.2) and Landsat data (NASA Landsat Program, 2006, Landsat ETM+ scenes 218–312 and 218–300, Orthorectified, USGS, Sioux Falls, 03/05/2006 and 04/15/2006, respectively; http://glcfapp.glcf.umd.edu:8080/esdi/index.jsp).

**Figure 2:**
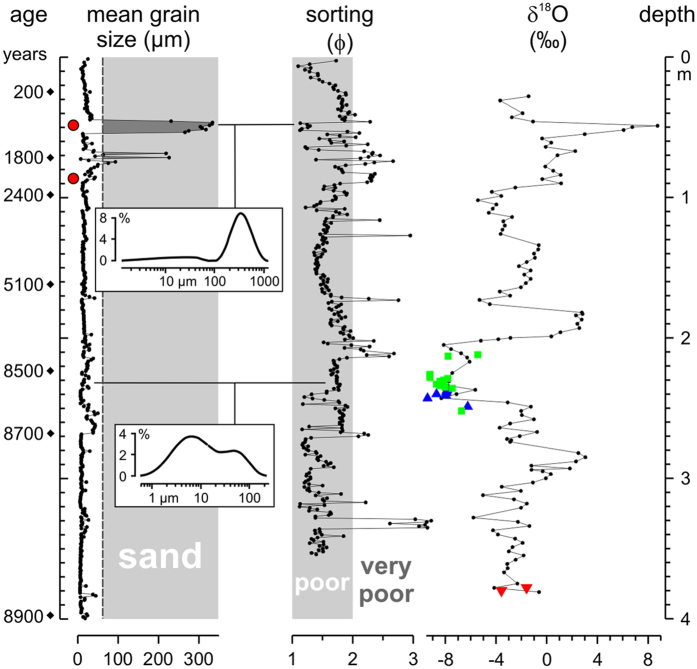
Grain size and stable isotope data for the pit section (upper 4 m) in the Lop Nur depression. Grain size frequency distributions shown for sand- (upper) and silt-dominated (lower) sediments. Red dots indicate position of samples used for grain-shape analysis (Fig. S1). δ^18^O values for bulk carbonate and ostracod shells (green square – *Limnocythere inopinata*, blue triangle – *Eucypris mareotica*, red inverted triangle – *Cyprideis torosa*). Age data of optically stimulated luminescence dating at left^19^.

The intensification of farming was at least locally accompanied by an inappropriate use of water resources which resulted in decreasing natural vegetation, mobilization of aeolian sand, and soil and water salinization^13^,^14^. Accordingly, shortage of water and desertification of oases in the Hexi Corridor was reported for a period ca. 2100 years ago^13^. Sanctions against the felling of trees to combat desertification in the Loulan Kingdom were reported for the 3^rd^ century CE^21^; but such evidence for desertification as a result of inappropriate use of water resources during the Han Dynasty is generally rare. However, the systematic assessment of geographical information available in historical documents and literature from the Han Dynasty shows that the farmland area was significantly larger upstream of Lop Nur and around Bosten Lake than it was in the last 400 years^5^. Remnants of extensive irrigated fields and irrigation channels from the Han Dynasty have been discovered near Loulan and in the Ruoqiang region^12^,^22^,^23^ (Fig. 1). Thus, water withdrawal for irrigation farming in the middle reaches of the Tarim and Konqi Rivers must have occurred on a large scale and probably caused water shortage in the lower reaches and declining Lop Nur lake levels.

In contrast to changing climate conditions, the decline of the Loulan Civilization and abandonment of the desert cities may have resulted from the intensification of irrigation farming in the middle reaches of the Tarim River and its tributaries and water shortage further downstream. Other regional lake records from within and outside the realm of the Han Empire potentially provide a clue to differentiate between the regional climate conditions and possible human impact on the environment during the period of the Loulan Kingdom decline.

**Figure 3:**
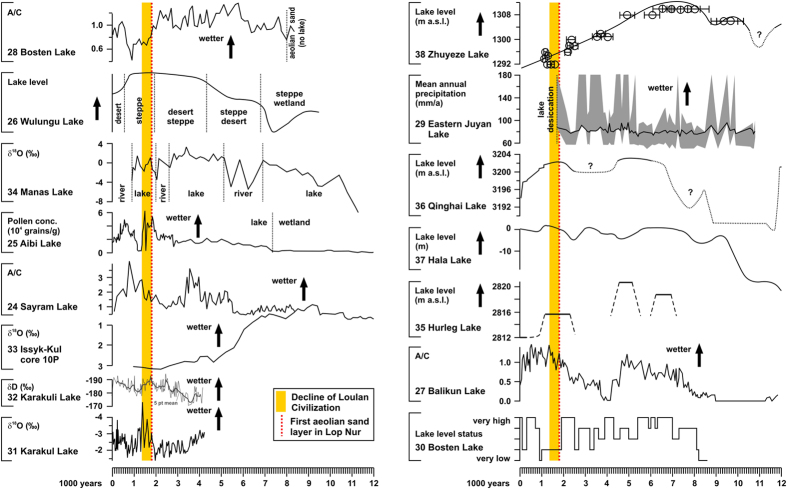
Comparison of climate inferences for the last 12,000 years based on lake records from Central Asia. Data are arranged to show wetter conditions towards the top. Lake numbers refer to information source in reference list.

**Figure 4:**
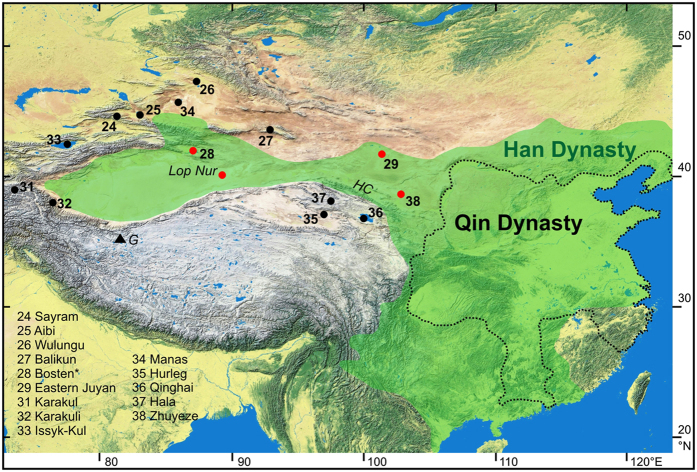
Positions of lake records within the Han Dynasty (206 BCE–220 CE) realm (green shading Han area) and outside. Lake level declines or periods of desiccation during the decline of the Loulan Kingdom were recorded at lakes indicated by red circles. The dotted line marks the realm of the preceding empire of the Qin Dynasty (221–206 BCE). Lake numbers refer to information source in reference list (*information for Bosten Lake also provided by ref. 30; HC – Hexi Corridor, G – Guliya ice core). Background map generated using a freely available map (https://commons.wikimedia.org/wiki/File:China_topography_full_res.jpg) and CorelDRAW version 12 (http://www.coreldraw.com/).

Pollen preserved in lake sediments indicate relatively wet conditions in the 3^rd^–7^th^ century in the north of Lop Nur at lakes Sayram^24^, Aibi^25^, Wulungu^26^ and Balikun^27^ (Figs 3 and 4). In contrast, a drop in the *Artemisia*/Chenopodiaceae (A/C) moisture index at Bosten Lake^28^ around 1800 years ago and the desiccation of the Eastern Juyan Lake at ca. 1700 years^29^ indicate drier conditions. Drier conditions at Bosten Lake were also inferred from geochemical data suggesting a drop of the lake level at ca. 1900 years ago^30^. Stable oxygen isotope data of lake carbonates or hydrogen isotopes of alkanoic acids indicate relatively wet conditions for lakes in the west^31^,^32^,^33^ and north^34^ of Lop Nur (Figs 3 and 4). The level of Issyk-Kul in the Tienshan Mountains was generally low during the late Holocene in comparison to the early Holocene but the slight δ^18^O decrease after 1800 years was assigned to a lake level highstand 16 m above the present level^33^. Lake Manas further in the northeast experienced a shift from a fluvial depositional setting to a lake environment ca. 2000 years ago^34^, providing further support for relatively wet conditions (Figs 3 and 4). The levels of Hurleg^35^, Qinghai^36^ and Hala Lake^37^ in the southeast of Lop Nur were relatively high in the 3^rd^–7^th^ century whilst those of Zhuyeze Lake^38^ declined (Figs 3 and 4). The resulting spatial distribution of lake conditions in the 3^rd^–7^th^ century shows low levels or even desiccation for lakes Lop Nur, Bosten, Eastern Juyan and Zhuyeze (Fig. 4). Relatively wet conditions were recorded at all other locations. This pattern corresponds to lowered lake levels or lake desiccation within the Han Empire realm, and relatively wet conditions adjacent to this region. Thus, regional climate conditions were generally relatively wet during the Loulan Kingdom decline. The inference of wetter conditions in western China in the 3^rd^–7^th^ century is also supported by higher ice-accumulation rates on the Guliya ice cap in the Kunlun Mountains south of the Tarim Basin^39^ (Fig. 4) and increased winter precipitation in the Kyrgyz Tianshan^40^. Thus, declining levels of the lakes Lop Nur, Bosten, Eastern Juyan and Zhuyeze most likely resulted from water withdrawal during the Han Empire intensification of irrigation farming along the mountain forelands. The decline of the Loulan Civilization was not a consequence of climate change but probably the result of a major man-made environmental disaster comparable to the recent Aral Sea crisis.

The reviewed lake records^24^,^25^,^26^,^27^,^28^,^29^,^30^,^31^,^32^,^33^,^34^,^35^,^36^,^37^,^38^ (Figs 3 and 4) and the ice core record from the nearby Kunlun Mountains^39^ indicate that climate conditions were relatively wet at the end of the Han Dynasty. If water withdrawal for irrigation farming in the mountain forelands of the Tarim Basin during the Han Dynasty caused the decline of the Loulan Civilization, a migration of ancient desert oases from more central positions towards the basin margins is expected over the Han period. Age data for sub-recent remains of vegetation and traces of human activities in the Tarim Basin will provide information about periods favourable for the growth of riverine vegetation and human activities. Thus, the lack of such age data only provides indirect evidence for less favourable conditions. Dating results for vegetation remains and remnants of settlements and agriculture in the Tarim Basin are generally rare and insufficient for Loulan and the vicinity of Lop Nur, but dated organic materials from the lower sections of the Keriya and Niya Rivers allow first assessments of available data from two river systems in the central Tarim Basin^7^,^41^ (Fig. 1).

Radiocarbon age data were presented for organic remains from the lowermost 100 km of the Keriya River^7^. Organic remains (sheep manure, *Populus* stems from house or city wall remnants, chopped *Populus* stems) representing direct or indirect evidence for human activities show a tendency of older ages further downstream (as old as 2600 years) and younger ages (not older than 2000 years) upstream^7^ (Fig. S2). *Tamarix* roots and *Populus* stems recovered from river terraces possibly represent natural gallery forest vegetation of a desert river. The six available age data cluster in a wide range with oldest and youngest age data for the most distal positions of the Keriya River. The data for the *Tamarix* roots and *Populus* stems possibly indicate that natural vegetation was able to survive in favorable positions with near–surface groundwater levels. The small data set of 14 dated organic remains does not include materials with an age between ca. 1600 and 600 years (Fig. S2). The three age data younger than ca. 600 years possibly indicate that climate conditions and/or human impact were apparently not supporting sufficient runoff and vegetation growth in the Keriya River region until ca. 600 years ago.

Exact locations of radiocarbon dating samples from Niya River were not presented by the authors but samples were grouped into northern, middle and southern parts^41^. Remains of trees and wooden building materials from the ancient Niya site to the east of the Keriya River similarly provided oldest ages in its northern part (ca. 2400 years) and successively younger ages in its middle (ca. 1900 years) and southern parts (ca. 1500 years^41^; Fig. S3).

Ancient sites from both rivers in the central Tarim Basin indicate a migration of human activities upstream which could have resulted also from climate change. However, the implied migration of ancient desert oases towards more marginal positions in the Tarim Basin over the duration of the Han Dynasty is at least also consistent with our assumed shortage of runoff farther downstream as a result of water withdrawal for irrigation farming in the middle reaches of the rivers. Based on the decline of lake levels or even desiccation of lakes within the Han Empire realm in contrast to relatively wet conditions in adjacent regions, we conclude that the Loulan Civilization decline was caused by intensified consumption of water for irrigation farming upstream of the oases and represents an early man-made environmental disaster.

It is puzzling that pre-Han age data for organic remains from ancient sites along the courses of the Keriya and Niya Rivers are confined to the lowermost reaches (Figs S2 and S3). Apparently, settlements and land use prior to the Han Dynasty were restricted to the lowermost river sections. We only may speculate that hazardous flooding, frequent river-channel displacements and/or poorer edaphic conditions (coarser and less nutrient-rich sediments) inhibited the occupation of such locations as long as water was sufficient farther downstream.

It also remains unclear whether significantly older ancient sites further downstream in the Tarim Basin (Gumugou and Xiaohe in its eastern part, 4000–3500 years) and younger but mostly pre-Han sites at its margins (Zaghunluq and Sampula in its south, ca. 2700–2000 years) possibly represent a climate or man-controlled shift of water resources in northwestern China prior to the Han Dynasty, or whether this tendency is not representative due to insufficient data^42^,^43^ (Fig. 1).

## Materials and Methods

### Grain size analysis of Lop Nur section sediments

Sediment samples from the pit section in the Lop Nur Basin were used for grain size analysis at Peking University in Beijing. Samples were treated with 30% H_2_O_2_ at room temperature to remove organic material, with 10% HCl to remove carbonates and deflocculated using a solution of 0.01 M (NaPO_3_)_6_. Following each step, the sample solution was boiled to promote the removal of remaining organic matter or carbonates. After 3-min sonication in deionized water, the sample solution was analyzed using a Malvern Mastersizer 2000 grain-size analyzer with 0.015 μm resolution and a range of 0.002–2000 μm with a relative error of <1%. The mean grain size was calculated using the Malvern software (Ver. 5.12 C), and sorting was examined with GRADISTAT^44^.

### Grain shape examination of Lop Nur section sediments

Grain shape was analyzed for sediments from the pit section collected at two stratigraphic levels. One sample from 0.87–0.86 cm depth was regarded to represent the bulk of the silt-dominated sediments of the section and a second sample from 0.49–0.48 cm depth represents the coarser, sand-dominated sediments from the upper part of the section. Subsamples were treated with 3% H_2_O_2_ for 48 hours and washed through 100 and 250 μm sieves. Sand grains remaining on the 250 μm sieve were dried, evenly spread on a stub and examined using a Zeiss Supra 40 VP Scanning Electron Microscope (SEM) at Freie Universität Berlin. SEM images of the first 25 separately placed sand grains (excluding overlaying grains) were obtained.

### Stable isotope analyses of bulk carbonate and ostracod shells

Stable isotope analysis of bulk carbonate was carried out using a Finnigan MAT 252 isotopic ratio mass spectrometer (IRMS) at the Lanzhou Institute of Geology, Chinese Academy of Sciences. Bulk samples were powdered and baked at 300 °C for 1 hour in a vacuum system, before the samples were reacted with 100% H_3_PO_4_ at 90 °C. The produced CO_2_ was trapped in a cold finger with liquid nitrogen and transferred into the mass spectrometer for the stable isotope analysis. The reliability of bulk carbonate for stable isotope analysis was tested at stratigraphic sections containing ostracod shells. Ostracod shells form within a few hours or days during moulting and reflect the stable isotope composition of host waters biased by a vital effect of commonly 1–2‰^45^,^46^. For stable isotope analysis of ostracod shells, sub-samples were treated with 3% H_2_O_2_ for 48 hours and washed through 100 and 250 μm sieves. Ostracod shells were picked from the >250 μm sieve residues. On average, 50 μg of shell material (11 shells of *Limnocythere inopinata*, six shells of *Eucypris mareotica*, two shells of *Cyprideis torosa*) was reacted with 103% H_3_PO_4_ at 70 °C in an automated carbonate preparation device (KIEL IV) coupled to a Finnigan MAT 253 IRMS at the German Research Centre for Geosciences in Potsdam. Isotopic ratios are given in delta notation relative to VPDB (Vienna Pee Dee Belemnite) calibrated with NBS-19 (δ^18^O = −2.20‰). The analytical precision is better than ±0.06‰ for the ostracod shell and ±0.3‰ for the bulk carbonate samples (1σ).

### Review of lake record data

Available lake records from northwestern China and adjacent regions were examined to distinguish between climate change presumably affecting a larger region and human impact expected to be higher in the realm of the Han Dynasty in comparison to the regions outside the Chinese Empire. Inferences from the lake records are based on four different types of evidence: (1) pollen data (six records), (2) stable isotope data (four records), (3) directly dated shorelines above the present lake level (three records), and (4) the sedimentological and geochemical characteristics of lake sediments (two records).

(1) Pollen data were presented for the Lakes Sayram^24^, Aibi^25^, Wulungu (Ulungur^26^), Bosten^28^, Balikun^27^ and the Eastern Juyan Lake^29^. The *Artemisia*/Chenopodiaceae ratio was used as climate proxy for the records from Sayram, Bosten and Balikun with higher ratios indicating steppe vegetation and wetter conditions in the arid to semi-arid region of Central Asia^47^. Higher pollen concentrations in the lake sediments were used as a proxy of higher vegetation density and wetter conditions around Lake Aibi^25^. Pollen and geochemical data of lake sediments were both assessed for Lake Wulungu and used to reconstruct climate conditions in the region and the lake level history^26^ which was later on confirmed through salinity and lake level inferences based on the distribution of micro-crustaceans in the modern and Holocene sediments of Lake Wulungu^48^. Holocene pollen data for the Eastern Juyan Lake were compared with modern pollen assemblages and used for a quantitative reconstruction of past precipitation in the region^29^. The lake desiccated 1700 years ago and aeolian sands were accumulated in the former lake basin^29^.

(2) Stable isotope records were provided from Lakes Karakul^31^, Karakuli^32^, Issyk-Kul^33^ and Manas^34^. The Lakes Karakul, Issyk-Kul and Manas are closed basin lakes; and δ^18^O values of lake carbonates represent the general hydrological budget of the lake with higher δ^18^O values indicating more arid conditions and lower values representing more humid conditions^49^. Lake Karakuli is an open-basin lake and lower δD values for plant leaf wax from the lake sediments were interpreted to indicate cooler and wetter climate conditions^32^.

(3) Ancient shorelines above current lake levels were dated at Lakes Hurleg^35^, Qinghai^36^ and Zhuyeze^38^, providing direct evidence for the lake level changes and climate conditions in the past. Optically stimulated luminescence dating was applied at these three lakes due to the absence of organic matter commonly used for radiocarbon dating of Holocene lake deposits. The obtained age data of the shorelines are generally in good agreement with independent lake-level inferences based on analyses of sediments from these lakes^50^,^51^,^52^.

(4) Sedimentological and geochemical proxies such as grain size, carbonate and total organic carbon content, and Sr/Ca ratios (a salinity and indirect lake-level proxy) were used for lake-level inferences from Lakes Bosten^30^ and Hala^37^. Pollen data are available for Lake Bosten and a comparison of the lake level inference based on the sedimentological and geochemical characteristics of the sediments and the A/C ratio shows a good agreement especially for the last 2500 years relevant for this study (Fig. 3).

### Data availability

The presented data are accessible in the PANGAEA database, https://doi.pangaea.de/10.1594/PANGAEA.871173.

## Additional Information

**How to cite this article:** Mischke, S. *et al*. The world’s earliest Aral-Sea type disaster: the decline of the Loulan Kingdom in the Tarim Basin. *Sci. Rep.* **7**, 43102; doi: 10.1038/srep43102 (2017).

**Publisher's note:** Springer Nature remains neutral with regard to jurisdictional claims in published maps and institutional affiliations.

## Supplementary Material

Supplementary Figures
