# Supplementary material for: The world’s earliest Aral-Sea type disaster: the decline of the Loulan Kingdom in the Tarim Basin
Source: Sci Rep. 2017 Feb 27;7:43102. doi: 10.1038/srep43102 (PMC5327390; doi:10.1038/srep43102)
Supplement: Supplementary Figures [file srep43102-s1.doc]

**Supplementary Information for:**

The world’s earliest Aral-Sea type disaster: the decline of the Loulan Kingdom in the Tarim Basin

Steffen Mischke^a1^, Chenglin Liu^b^, Jiafu Zhang^c^, Chengjun Zhang^d^, Hua Zhang^b^, Pengcheng Jiao^b^, Birgit Plessen^e^

^a^Faculty of Earth Sciences, University of Iceland, 101 Reykjavík, Iceland

^b^Institute of Mineral Resources, Chinese Academy of Geological Sciences, Beijing 100037, China

^c^MOE Laboratory for Earth Surface Processes, Department of Geography, College of Urban and Environmental Sciences, Peking University, Beijing 100871, China

^d^School of Earth Sciences and Key Laboratory of Mineral Resources in Western China, Lanzhou University, Lanzhou 730000, China

^e^Helmholtz Centre Potsdam, German Research Centre for Geosciences, Potsdam 14473, Germany

^1^To whom correspondence should be addressed. Email: smi@hi.is.


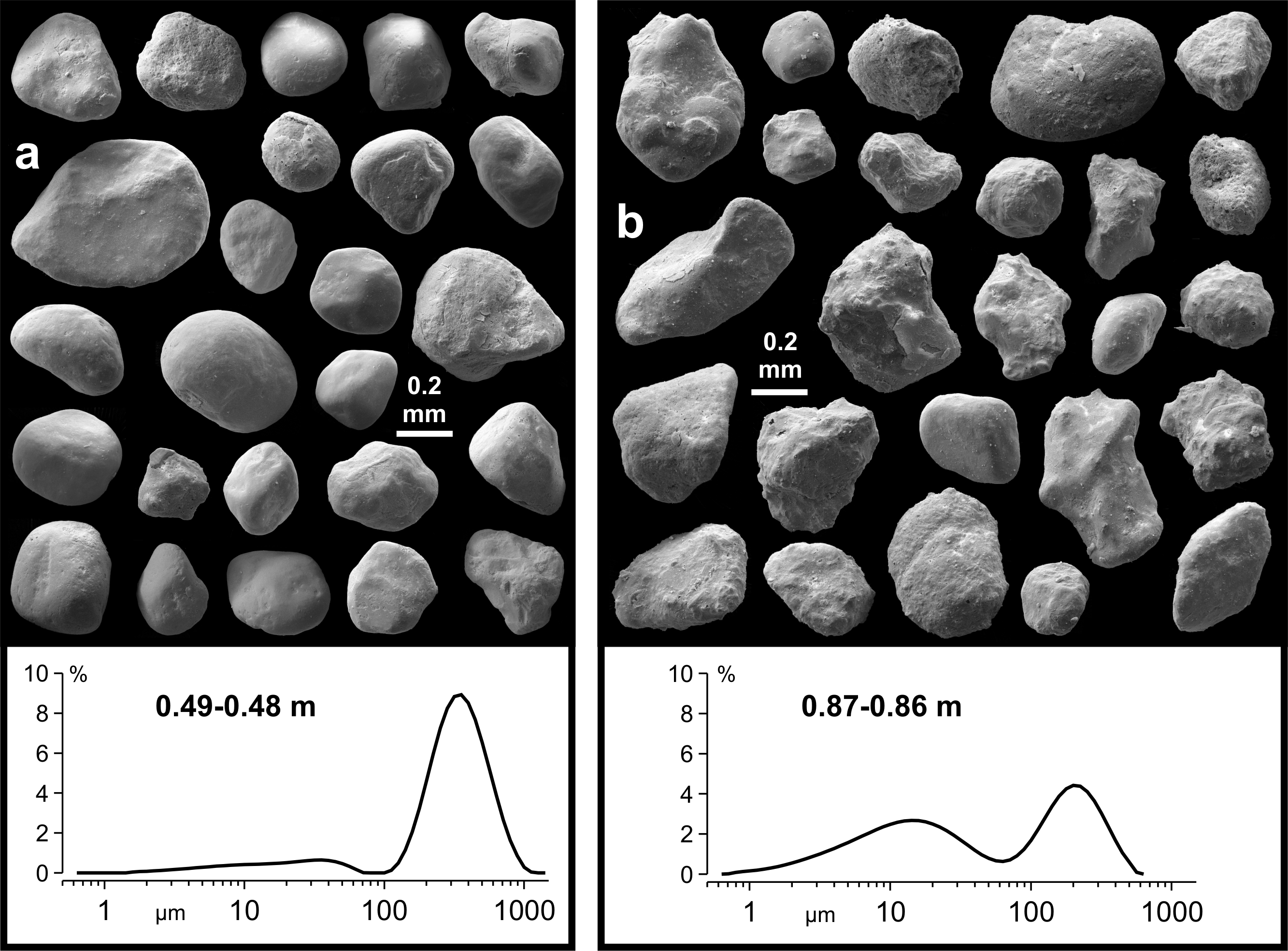


Fig. S1

Grain shapes for sand grains from the pit section in the Lop Nur Basin. a, Sand grains from 0.49-0.48 m depth in the section representing mostly aeolian sand, and grain-size frequency curve. b, Sand grains and frequency curve for sediments from 0.87-0.86 m representing the silt-dominated sediment which was probably mostly transported to the lake by the inflowing rivers.


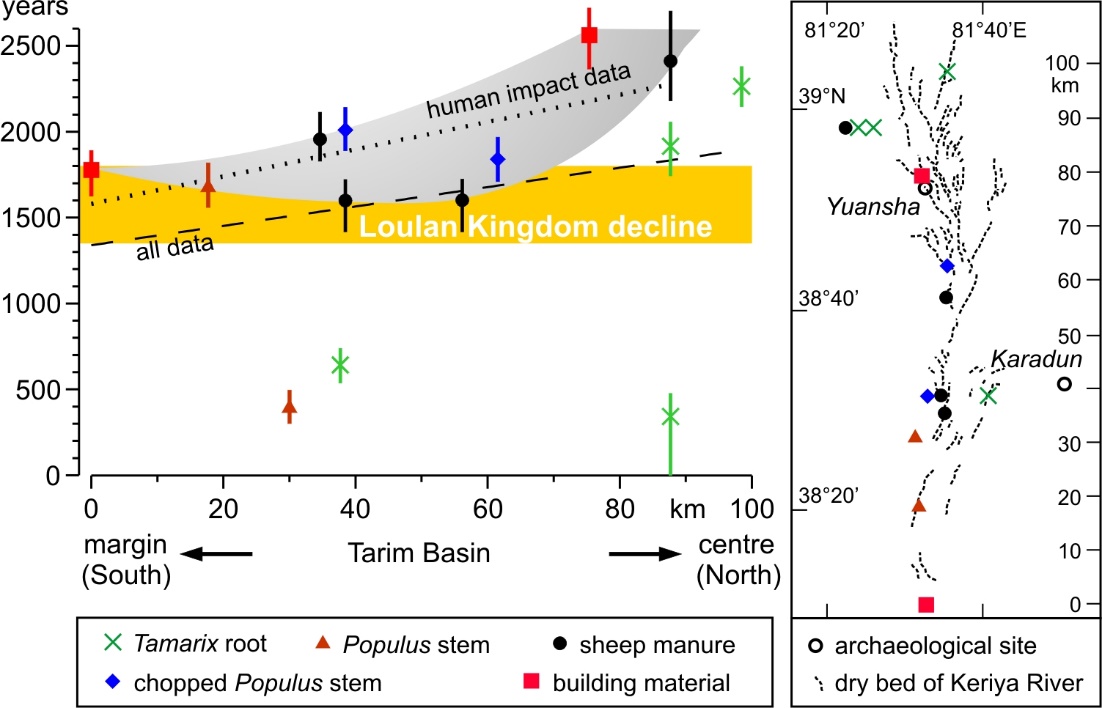


Fig. S2

Age data for organic remains from the lower Keriya River (7; median and 2σ probability ranges) arranged along flow direction from south to north. Materials providing direct and indirect evidence for human activity are arranged in the grey shaded region. The broken and dotted lines represent linear regressions for all data and those providing evidence for human activities, respectively. The period framing the Loulan Kingdom decline is indicated by the orange bar. The right panel shows a map of the dry Keriya River bed and the sampled locations (7). Published radiocarbon age data were calibrated as calendar years with 2 probability range using OxCal 4.2 and IntCal13 (52-53). Map in right panel generated with CorelDRAW version 12 (<http://www.coreldraw.com/>). The locations of dry river beds and sample sites were displayed in a slightly simplified way based on the original sketch map of the cited publication.


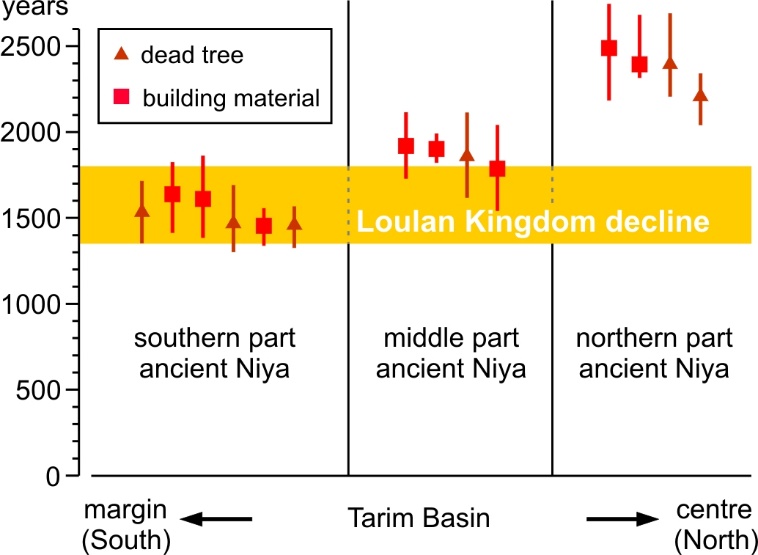


Fig. S3

Age data for organic remains from the lower Niya River (41; median and 2σ probability ranges) arranged along flow direction from south to north. The period framing the Loulan Kingdom decline is indicated by the orange bar. Published radiocarbon age data were calibrated as calendar years with 2 probability range using OxCal 4.2 and IntCal13 (52-53).

**References:**

52. Bronk Ramsey, C., Lee, S. Recent and planned developments of the program OxCal. *Radiocarbon* **55**, 720-730 (2013).

53. Reimer, P.J., et al. IntCal13 and Marine13 radiocarbon age calibration curves 0-50,000 years cal BP. *Radiocarbon* **55**, 1869-1887 (2013).
